# EGT-UNet: Evolutionary Game-Theoretic Adaptive Optimization for Pediatric Panoramic Tooth Segmentation

**DOI:** 10.3390/bioengineering13070840

**Published:** 2026-07-21

**Authors:** Muhammet Emin Sahin, Hasan Ulutas, Halil I. Cosar, Tayyip Bicer, Recep B. Gunay, Süleyman K. Buyuk

**Affiliations:** 1 Queen Mary’s Digital Environment Research Institute (DERI), London E11HH, UK; 2 Department of Computer Engineering, Izmir Bakircay University, 35665 Izmir, Türkiye; 3 Department of Computer Engineering, Yozgat Bozok University, 66100 Yozgat, Türkiye; 4 Department of Electrical and Electronics Engineering, Yozgat Bozok University, 66100 Yozgat, Türkiye; 5 Department of Orthodontics, Faculty of Dentistry, Ordu University, 52200 Ordu, Türkiye; 6 Department of Computer Technologies, Yozgat Bozok University, 66700 Yozgat, Türkiye; 7 Department of Dental Biomaterials Science, Seoul National University, Seoul 03080, Republic of Korea

**Keywords:** pediatric tooth segmentation, panoramic radiography, deep learning, EGT-UNet

## Abstract

The present work proposes EGT-UNet, an innovative loss optimization mechanism based on Evolutionary Game Theory (EGT) aimed at pediatric panoramic tooth segmentations. In the absence of a large set of high-quality images in the current literature, we developed a novel pediatric panoramic image database, including 1269 images from Ordu University Faculty of Dentistry. The dataset includes subjects aged 3–14, where 67% are males and 33% are females. The annotation of all images was done at the pixel level by a professional orthodontist with a two-fold verification process. In order to prevent data leakage, the dataset was split at the subject level into training/validation (85%) and independent test (15%) sets. Data in the training set were divided into folds for five-fold cross-validation. From a structural point of view, EGT-UNet is an improved version of U-Net, equipped with the following modules: Squeeze-and-Excitation blocks, Attention Gates, and a dilated convolutional module mimicking Atrous Spatial Pyramid Pooling. The main novelty of the proposed approach involves the application of the dynamic change in loss weights. Specifically, in our work, the fusion of three losses—Dice, Focal Tversky and Boundary—was dynamically tuned via replicator dynamics using task-specific fitness functions. The difference with traditional loss-weighting methods is that in the latter case, fixed weights are applied. To evaluate the effect of each component of the EGT-UNet, we conducted an ablation study of six architectures varying in terms of hybrid loss function and dynamic/static weight tuning. Using the independent test dataset, we observed a similar performance level of all models with Dice scores ~0.93. Our best model, called “EGT Aggressive”, achieved Dice = 0.931 ± 0.044, IoU = 0.873 ± 0.066, and Boundary Dice = 0.631 ± 0.064. Importantly, this model demonstrated a statistically significant superiority over the baseline network according to region-related metrics and boundary metrics (Wilcoxon *p* < 0.05). In computational studies, we revealed an increased model robustness when dealing with highly complex mixed dentition images.

## 1. Introduction

Regarding dental care services, the accuracy of treatment planning procedures is largely defined by the accuracy and efficiency of the segmentation procedure aimed at identifying anatomical structures. Tooth segmentation will be a crucial part of creating individualized dental models with the help of machine learning algorithms. Specifically, during tooth segmentation, teeth are isolated from the other anatomical structures in digital images to develop computerized models of oral cavities [1,2,3]. Processing data obtained via panoramic radiography (OPG), cone-beam computed tomography (CBCT), and intraoral scanning (IOS) can be tedious and require the involvement of professionals. Thus, introducing AI techniques for tooth segmentation can contribute to more efficient and uniform task execution [4]. Even though OPGs and bitewing radiographs are two-dimensional images, they have numerous applications in dentistry due to their wide field of view, low radiation dose, and accessibility. Most studies focus on employing convolutional neural networks (CNNs) and Transformer models for tooth segmentation. Hou et al. [5] improved U-Net models via adding attention and hybrid self-attention blocks to address blurred boundaries and low contrast in panoramic radiographs. This model demonstrated 98.53% accuracy. According to Mohan et al. [6], preprocessing the images via Otsu’s thresholding algorithm before applying the VGG-UNet model helped them to improve the overall segmentation quality with an accuracy of 98.84%. It is worth noting that U-Net is not sufficient for solving segmentation problems related to the existence of absent teeth. However, Nader et al. [7] managed to improve the model’s performance by up to 5–10% via the addition of a bounding box prior to the skip connections. As for instance segmentation tasks when teeth should be numbered and separately segmented, Mask R-CNN is preferable. For example, using this model, Yaren Tekin et al. [8] obtained a mean Average Precision (mAP) equal to 97.49% in tooth segmentation on bitewing radiographs. Similarly, by applying “collaborative learning” with Mask R-CNN and Faster R-CNN, Chandrashekar et al. [9] were able to increase the accuracy of tooth segmentation in panoramic radiographs to 98.77% (96% originally). Gardiyanoğlu et al. [10] applied U-Net on a large dataset and successfully segmented not only the teeth but also seven other anatomical structures (crown, implant, caries) with Dice Similarity Coefficient (DSC) scores of 85–94%.

Transformer models that excel in identifying global dependencies in images have also been employed in some studies. Sheng et al. used SWin-Unet, an exclusively Transformer-based model inspired by the U-Net structure [11], showing superiority over standard CNN models such as U-Net. Three-dimensional imaging offers advantages over two-dimensional imaging in terms of more accurate visualization of anatomical structures. In particular, CBCT is considered the gold standard in three-dimensional assessment of teeth, bone structures, and pathologies in adult and pediatric patients. The authors of a paper by Shaheen et al. [12] developed a three-step 3D U-Net model that is capable of fully automatic tooth segmentation and classification based on CBCT images and operates at least 1800 times faster than a human. Kirnbauer et al. [13] suggested a two-stage approach to detect periapical lesions that involves first locating the teeth and then segmenting them (SpatialConfiguration-Net followed by U-Net). To solve the problem of extremely computationally costly 3D data processing, Shen et al. [14] developed Grouped Bottleneck Transformer, a model that integrates CNNs and Transformer techniques. Wang et al. [15] proposed Trans-VNet, which combines a V-Net and Transformer architecture and incorporates cross-attention modules. Additionally, they used patch-based ROI extraction to minimize the effects of metal artifacts. In the meantime, Li et al. [16] designed a semantic graph attention (SGA) module to model anatomical relations among teeth, which helps decrease misclassifications of adjacent and symmetric teeth. Fontenele et al. [17] evaluated the effect of dental fillings on segmentation performance and showed that fillings slightly decrease the accuracy, but the AI tool remains clinically feasible (IoU >91%). Elsonbaty et al. [18] tested CNN-based platform for pediatric CBCT images for primary teeth segmentation. They reported 98% segmentation accuracy (95% Dice), 35 times higher speed compared to manual segmentation, and an increased number of segmented teeth. Mladenovic et al. [19] analyzed two commercial tools for CBCT tooth segmentation in a rare case involving nine supernumerary teeth belonging to a child and noted that one tool is capable of automatic detection of abnormalities while another one still requires a manual correction. Three-dimensional mesh and point cloud data from IOS provide great visualization opportunities and allow images to be obtained without exposure to ionizing radiation. Thus, they are widely used in orthodontics and prosthodontics. Recently, Graph Neural Networks (GNNs) and similar neural architectures have been developed to work with these kinds of non-Euclidean data. Thus, Wang et al. [20] proposed a 3D U-Net model for segmentation of IOS images even in difficult conditions (presence of orthodontic brackets, partial edentulism) with an IoU of 91%. Applying Dynamic Graph CNN (DGCNN), Im et al. [21] showed that their automatic segmentation (AS) algorithm yields 97.26% success rate and much shorter processing time compared to a commercial software. Some advanced GNN architectures are also discussed. In particular, Zhao et al. [22] applied a Graph Attentional Convolution (GAC) model to learn both local and global geometric features. TeethGNN is a GNN-based model that includes dual branches: segmentation label and offset-to-centroid prediction, which enables effective tooth isolation [23]. Lin et al. [24] proposed DBGANet, which includes branches for 3D coordinates and normals. Therefore, it allows detection of the tooth–gum boundary. Finally, Zhuang et al. [25] proposed a novel hybrid learning strategy that uses AlignNet to automatically align 3D models of arbitrary orientation received from different devices. In general, tooth anatomy of children is more complicated and dynamic than that of adults due to the presence of mixed dentition, which means that there are primary teeth, erupting permanent teeth, and tooth germs in pediatric cases. Asci et al. [26] conducted caries segmentation of 6075 panoramic radiographs of pediatrics and analyzed the performance in terms of primary, mixed, and permanent dentition stages separately. Although performance decreased in the case of the mixed dentition stage, this segmentation was still rather promising with an F1 score of 86.75%. Using Mask R-CNN, Rubiu et al. [27] achieved an accuracy of 98.4% and Dice score of 0.87 when segmenting 52 different categories (including primary and permanent teeth). Zhong et al. [28] proposed a model named GCNet that has attention mechanisms adapted to challenges posed by pediatric panoramic radiography (noise, blurred boundaries). It resulted in Dice scores with 0.9338. A challenge posed by this topic is insufficiently annotated pediatric datasets. That is why Zhang et al. [29] created a publicly available dataset consisting of panoramic radiographs of pediatric patients aged 2 to 13 years. Among the tested models, the standard U-Net achieved a mean IoU of 0.8387 and an accuracy of 97.10%.

Despite remarkable advances brought by deep learning techniques to tooth segmentation, most studies are focused on adult panoramic radiography obtained under clinical conditions. Pediatric panoramic radiographs (3–14 years old) face certain difficulties such as anatomical variation, mixed dentition, motion artifacts, and immature mineralization. This research proposes a novel deep learning model for automated tooth segmentation in pediatric panoramic radiographs. First of all, pediatric images are characterized by peculiarities, including mixed dentition, incompletely developed roots, overlapping anatomical structures, and low-contrast boundaries. To solve these problems, we assembled a dedicated dataset of manually annotated pediatric panoramic radiographs and developed an improved U-Net architecture with channel attention, spatial attention, and multi-scale contextual modeling mechanisms. However, in this paper, we do not limit ourselves to model architectural improvements but additionally offer an Evolutionary Game Theory (EGT)-based dynamic loss optimization scheme. Contrary to static allocation of loss weights, it allows dynamic reallocation of loss optimization priorities depending on fitness scores, which makes the algorithm adaptive to context changes. Comprehensive ablation studies on an independent test dataset have been performed. The key contributions of this paper are as follows:

- A dataset with 1269 panoramic radiographic images (children aged 3–14) was assembled, with pixel-wise annotation achieved with high accuracy through a two-step validation process involving domain experts, which resolves the problem of lack of annotated pediatric dental data.
- The base version of the U-Net architecture was further improved by adding Squeeze-and-Excitation blocks, Attention Gates, and an ASPP (Atrous Spatial Pyramid Pooling)-inspired multi-scale module.
- An innovative loss-weighting scheme based on the Evolutionary Game Theory (EGT) of adaptive evolution is developed to achieve dynamic trade-off between Dice, Focal Tversky, and Boundary losses using replicator dynamics.
- A systematic ablation experiment including six different models was performed, and statistical significance was assessed using paired Wilcoxon tests as well as effect-size analysis on an independent test dataset.
- The performance of segmentation algorithms was analyzed for different levels of structural complexity, and it was found that adaptive evolution of loss functions can be advantageous for more complex pediatric cases.

## 2. Material and Methods

### 2.1. Dataset

**Figure 1:**
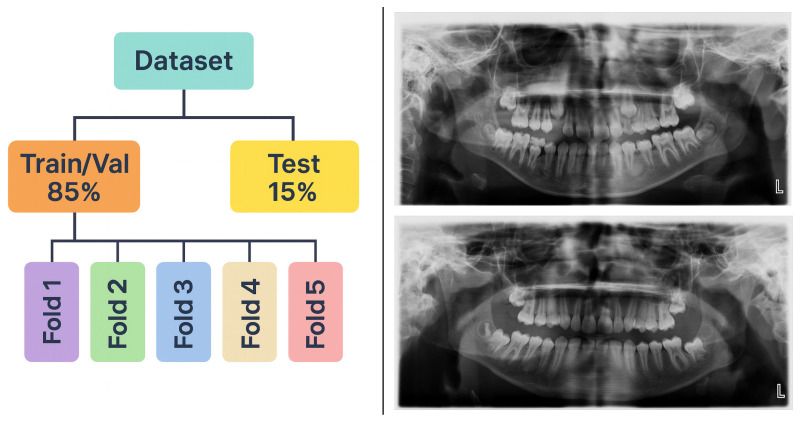
Overview of the dataset split and sample radiographs.

The dataset used in this work was gathered based on 1269 panoramic radiography images of pediatric dentition from the Dentistry Faculty of Ordu University. In the selected samples, the age of the patients ranged between 3 and 14 years, and 67% of them were males and 33% females [30]. Despite acquiring around 1500 panoramic radiography images, only those with high quality without any motion artifacts, and with full anatomical information, were chosen. The nature of pediatric dental imaging involves increased anatomical variance and reduced contrast of structural components, which explains the lack of annotated databases in the literature. To overcome the said challenge, each image was annotated by an orthodontist who manually annotated the image by identifying the pixels associated with dental structures and implementing two-stage validation. After initially selecting images without motion artifacts, any missing parts of the region of interest, or low contrast levels, the selected images were then split into 85% as training/validation sets and 15% as testing sets to prevent any data leakage during training. Overview of the dataset split and sample radiographs are shown in Figure 1.

To begin with, all image–mask pairs were manually validated to check whether there was correct correspondence between them. Afterward, 15% of the data was selected as an independent test set that remained untouched throughout all experiments. The remaining 85% of the dataset was divided into five parts based on the principle of 5-fold cross-validation. Each fold consisted of separate training and validation folders. This way, by creating different folders for every fold separately, each model was being trained on identical training and validation samples for this fold. Therefore, any possible discrepancies can be attributed only to the architecture and not to the data distribution scheme.

### 2.2. Data Preprocessing and Augmentation

For the purpose of maintaining consistency and increasing the generality of the model across various instances in pediatrics, a unified preprocessing protocol was used for all the images. First, all the images were resized into a common resolution of 256 × 256 pixels and normalized within an intensity range of [0, 1]. To boost local contrast and highlight minor morphological structures, the Contrast-Limited Adaptive Histogram Equalization (CLAHE) technique was used. Then, Gaussian blurring was performed to reduce noise without sacrificing any anatomical structure information. As an additional measure to prevent overfitting, a data augmentation procedure involving randomized modifications such as random rotation, flip, and changes in translation, scaling, and brightness-contrast was used.

### 2.3. Segmentation

Tooth segmentation is the separation of pixels pertaining to teeth from an image. It forms an essential part of computer-assisted dental analysis. Since its development by Ronneberger et al. [31], segmentation using deep learning models has been the most popular method, showcasing the success of encoder–decoder models in biomedicine. Some major developments in this regard are the V-Net architecture proposed by Milletari et al. [32] and the UNet++ algorithm developed by Zhou et al. [33]. These methods have enhanced multi-scale feature extraction and tooth boundary detection capabilities. Regarding dentistry, several recent works have confirmed the efficiency of automated tooth segmentation in improving the diagnostic process and facilitating quantitative analysis of the teeth. The CNN models have exhibited higher accuracy in comparison with other segmentation strategies such as thresholding and region-based methods. However, segmenting the teeth in the panoramic X-rays of children is a difficult task, primarily because of mixed dentition, incomplete root formation, and poor contrast in the enamel areas.

#### Problem Definition

The task of segmenting the teeth in panoramic images is considered in this paper as a binary semantic segmentation problem in which the target is to predict whether each pixel *p* ∈ Ω belongs to the tooth or background, i.e., *y* = 1 or *y* = 0, respectively. Pediatric panoramic imaging suffers from high variability due to a mixture of dentition, the stage of eruption, and insufficient mineralization. As a result, this leads to a lack of contrast at the boundaries and overlapping anatomy, making edge-based segmentation and region-based segmentation difficult. Deep learning models, including deep convolutional encoder–decoder networks such as U-Nets, have achieved great success in medical image analysis problems where segmentation is involved at the pixel level.

(1) $$f_{\theta }:X\to Y$$

Let us represent the input image as *X* and the predicted segmentation as *Y*, optimizing the problem through supervised training with ground-truth labels provided by medical experts. The main difficulty in this regard stems from the requirement to locate the boundaries accurately despite the presence of noise and structural uncertainty.

### 2.4. Proposed Model: EGT-UNet Architecture

The EGT-UNet model has been designed to tackle the unique challenges faced by pediatric panoramic X-ray images, which include mixed dentition, poor contrast, and overlap of anatomical structures.

#### 2.4.1. Baseline U-Net

The proposed segmentation model structure is based on the original U-Net design developed by Ronneberger et al. [31]. The U-Net architecture follows the symmetric encoder–decoder pattern and implements a fully convolutional network (FCN). It allows efficient combination of spatial information with semantic features by means of skip connections. In the encoder branch, there are four levels of downsampling blocks. They include two subsequent 3 × 3 convolution layers. Each of them is followed by Batch Normalization (BN) and Rectified Linear Unit (ReLU) to ensure stable learning and introduce non-linearity to the learning algorithm. Dimensionality reduction in the spatial domain is implemented using 2 × 2 max-pooling layers with a stride of two. Throughout this process, the number of feature channels grows gradually from 64 to 1024 up to the bottleneck (bridge) layer. The decoder branch represents four levels of upsampling layers that duplicate encoder blocks symmetrically. They use 2 × 2 transposed convolutions to expand the output tensor dimensions, followed by concatenation of encoder features through skip connections to recover possible loss of fine spatial details. Similar to the encoder branch, each decoder block utilizes two sequential 3 × 3 Convolution–BN–ReLU operations. Finally, the model output is produced by means of 1 × 1 convolution with Sigmoid activation to obtain a probability map for the segmentation mask prediction.

#### 2.4.2. Improved U-Net (Attention + SE + ASPP)

Three key architectural modifications were applied to overcome some inherent drawbacks of the Baseline U-Net model and increase the power of the feature representation.

**Squeeze-and-Excitation (SE) blocks:** In order to implement channel-wise adaptive recalibration of features, SE blocks were implemented right after the output layer of each encoder block in line with Hu et al. [34]. An SE block comprises two major building blocks. The first one is called Squeeze and involves global average pooling for aggregating spatial information into a channel descriptor. The second step—Excitation—implies modeling of non-linear interactions between channels by applying two fully connected layers with a ratio of reduction equal to 16 and Sigmoid activation function to obtain channel coefficients that are then used to weight input features.

**Attention Gate (AG):** Following the concept proposed by Oktay et al. [35], Attention Gates were introduced to perform selective learning of important spatial locations within the skip connections.

(2) $$\alpha =\sigma \left(\psi^{T}(\mathrm{ReLU}(W_{g}g+W_{x}x+b_{g}))+b_{\psi }\right)$$

In this mechanism, a gating signal g, derived from the low-resolution feature maps of the decoder branch, is combined with the encoder feature maps x transmitted through the skip connections. Specifically, the two feature representations are first projected into a common embedding space via learnable linear transformations $W_{g}$ and $W_{x}$, and the resulting summation is passed through a ReLU non-linearity to introduce sparse, non-linear feature selection. A subsequent linear projection using the weight vector $\psi^{T}$ collapses the representation to a scalar map, which is then normalized to the range [0, 1] by a Sigmoid activation function σ, yielding the attention coefficient α as formalized in Equation (2). In the Equation, $b_{g}$ and $b_{\psi }$ are biases. This spatial attention map is applied multiplicatively to the encoder features before they are concatenated with the decoder activations, effectively amplifying task-relevant structures such as lesion boundaries and suppressing spurious background responses. By conditioning the attention on the high-level semantic context encoded in the gating signal, the model learns to localize salient regions in a data-driven manner without requiring external supervision, thereby improving both segmentation precision and computational efficiency.

**ASPP (Atrous Spatial Pyramid Pooling)-Motivated Dilated Convolution Block:** At the bridge level, a dilated convolution block that is based on ASPP was used to achieve better multi-scale context modeling [36]. In this block, multiple dilated convolutions with different dilation ratios such as *r* = 6 and *r* = 12 were used in parallel. Using this arrangement, an expansion of the receptive field was achieved without an increase in the parameter size. This multi-scale context extraction approach is especially useful for segmenting dental structures.

#### 2.4.3. Loss Functions

A novel loss function is designed in this paper in order to address both major problems of medical image segmentation at once. These include severe class imbalance and imprecise boundaries. The general loss function used is defined as follows: Dice loss ($L_{dice}$), Focal Tversky loss ($L_{focal}$), and Boundary loss ($L_{boundary}$). The combined loss is defined as

(3) $$\begin{array}{cccc} & L_{total}=w_{dice}\cdot L_{dice}+w_{focal}\cdot L_{focal}+w_{boundary}\cdot L_{boundary} & & \end{array}$$

where $w_{dice}$, $w_{focal}$, and $w_{boundary}$ represent the weight factors determining the contribution ratio of each component loss. In particular, Dice loss is utilized for optimizing the degree of overlap between the predicted segmentation and the ground-truth mask, which helps ensure global structure consistency and reduce volume bias. However, the utilization of Dice-based optimization alone might be inadequate in situations where there is an extremely large class imbalance. Therefore, Focal Tversky loss is included in the loss function for reweighing the contribution of each sample and enhancing the learning process through down-weighting easily classified samples and focusing on misclassified samples. In order to improve edge detection accuracy, the Boundary loss function is included in the model as a distance-based regularizer. In specific, the Boundary loss penalizes the gap between the predicted boundary and the corresponding ground-truth boundary in the space domain, thereby encouraging better edge detection.

**Dice Loss:** Dice loss is a differentiable surrogate of the Dice Similarity Coefficient (DSC), extensively adopted in medical image segmentation due to its robustness against class imbalance. It directly quantifies the spatial overlap between predicted probabilities and the ground-truth mask. The loss is formulated as:

(4) $$\begin{array}{cccc} & L_{dice}=1-\frac{2\sum (p\cdot g)+\epsilon }{\sum p^{2}+\sum g^{2}+\epsilon } & & \end{array}$$

Here, *p* stands for the probability map and g for the binary ground-truth mask, while ε is a small constant ($10^{-7}$) added to ensure numerical stability. Since Dice loss is an overlap-based score, it maximizes the overlap between two sets and becomes highly efficient for foreground-background class imbalance scenarios.

**Focal Tversky Loss:** To improve the results on hard and minority class cases, the Focal Tversky Loss was used in this study. The Focal Tversky Loss is based on the Tversky index (TI), which is the generalization of the Dice score and can apply unequal weighting to FP and FN by tuning parameters α and β.

(5) $$\begin{array}{cccc} & TI=\frac{TP+\epsilon }{TP+\alpha \cdot FN+\beta \cdot FP+\epsilon } & & \end{array}$$

The focal variant introduces a modulating parameter γ to emphasize hard-to-classify pixels:

(6) $$\begin{array}{cccc} & L_{focal\_tversky}=(1-TI{)}^{\gamma } & & \end{array}$$

In this study, the hyperparameters were empirically determined as α=0.7, β=0.3, and γ=0.75. Increasing the importance of α will place greater penalties on false negatives. In dental imaging, the importance of not missing a tooth anatomy is greater than that of predicting false positives. The second focal exponent, γ, makes optimization more sensitive to misclassified pixels, thus improving detection of small anatomical structures.

**Boundary Loss:** In order to detect boundaries with greater accuracy, we introduce Boundary loss. This loss term guides the optimization with respect to edges. These edges are computed using the gradient edge representation of the image, which is compared to the one from the ground-truth image. Using the Sobel filter E(·), the Boundary loss is defined as:

(7) $$\begin{array}{cccc} & L_{boundary}=\sum |E(p)-E(g)| & & \end{array}$$

**Figure 2:**
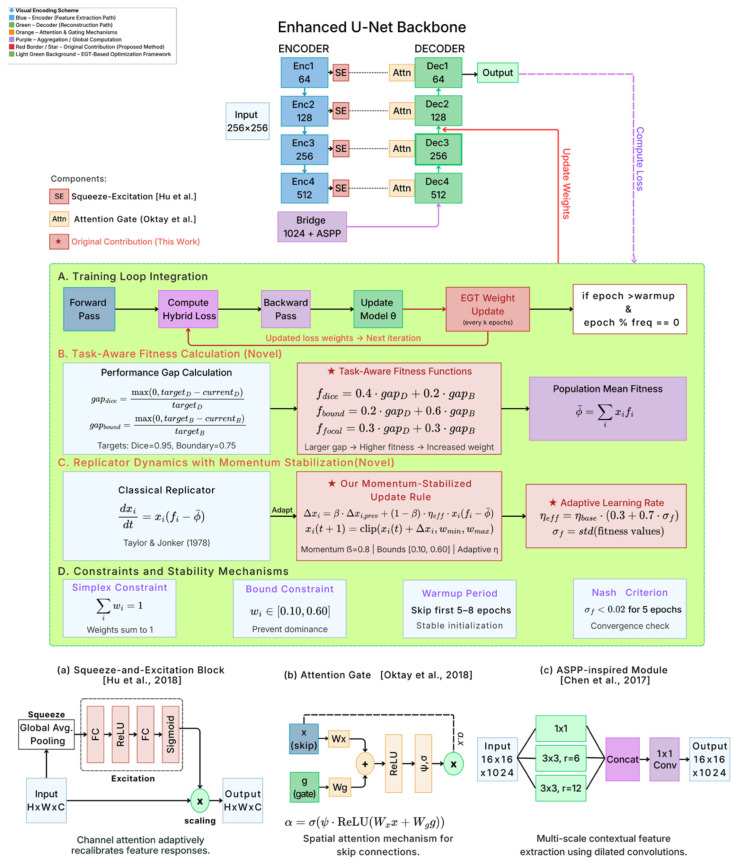
Detailed functional components of EGT-UNet.

Through penalties imposed on differences between predictions and reference edges, the formulation promotes sharper edge detection with greater spatial consistency. This optimization is particularly important when working within the context of medical processes like treatment planning and morphometric studies, where small variations in edge locations can lead to significant inaccuracies in diagnosis and treatment. Detailed functional components of EGT-UNet is given in Figure 2.

### 2.5. EGT-Based Dynamic Loss Optimization

One of the main innovative methodologies suggested in this paper is the application of adaptive loss-weighting based on Evolutionary Game Theory (EGT). As opposed to techniques utilizing fixed parameters to weigh different elements of the loss function, this technique makes use of adaptive weights obtained through replicator dynamics.

In this study, we utilize Evolutionary Game Theory because adaptive loss-weighting is naturally a constrained multi-objective problem on the probability simplex: the three weights must remain non-negative and sum to one (Equation (9)). Replicator dynamics solve exactly this problem, treating each loss term as a competing strategy and reallocating optimization pressure toward whichever objective currently underperforms its clinical target. Unlike grid or Bayesian searches over fixed weights, it requires no expensive offline search, and unlike gradient-magnitude heuristics it does not compare gradients of losses that live on very different scales (overlap-based Dice versus distance-based Boundary loss); it also provides bounded, simplex-constrained updates with an explicit stability (Nash) criterion.

#### 2.5.1. Evolutionary Game Formulation

To formalize the adaptive loss-weighting mechanism introduced in the preceding section, the multi-component loss optimization problem is cast as an evolutionary game. In this framework, each competing loss term is treated as a distinct player whose influence evolves over time based on its contribution to segmentation performance. Specifically, the problem is modeled as a three-player game, where each player corresponds to a specific objective:

- **Player 1 (Dice Loss):** Global structural consistency.
- **Player 2 (Focal Tversky Loss):** Class imbalance mitigation and hard-sample sensitivity.
- **Player 3 (Boundary Loss):** Contour precision and edge fidelity.

Each player’s strategy represents its relative contribution to the total loss. The strategy vector is defined as

(8) $$s=[w_{dice},w_{focal},w_{boundary}]$$

subject to the simplex constraint

(9) $$\sum_{i}w_{i}=1,0\leq w_{i}\leq 1$$

Within this framework, the evolution of weights is governed by their task-dependent fitness, allowing the system to adaptively reallocate optimization pressure according to current segmentation performance.

#### 2.5.2. Task-Aware Fitness Function

In order to account for the contribution from each part of the loss function, task-specific fitness criteria are defined. Fitness is evaluated not merely based on gradients, but rather based on discrepancies against clinical objectives:

(10) $$\begin{array}{cccc} & dice\_gap=\frac{max(0,target_{dice}-current_{dice})}{target_{dice}} & & \\ & boundary\_gap=\frac{max(0,target_{boundary}-current_{boundary})}{target_{boundary}} & & \end{array}$$

Here, the targets were empirically set to

(11) $$target_{dice}=0.95,target_{boundary}=0.75$$

The fitness values $f_{i}$ are constructed as weighted combinations of these gaps:

(12) $$f_{dice}=0.4\cdot dice\_gap+0.2\cdot boundary\_gap\;f_{focal}=0.3\cdot dice\_gap+0.3\cdot boundary\_gap\;f_{boundary}=0.2\cdot dice\_gap+0.6\cdot boundary\_gap$$

This design ensures performance-driven prioritization: when boundary quality deteriorates, the fitness of the Boundary loss increases, encouraging replicator dynamics to assign it greater influence in subsequent updates.

#### 2.5.3. Replicator Dynamics with Momentum Stabilization

Once fitness values for each loss component have been determined, the next step is to define how the strategy weights evolve over successive training iterations. To do this, we apply a discrete-time version of the classical replicator dynamics describing the evolution of each player’s strategy in time according to the rule

(13) $$\frac{dx_{i}}{dt}=x_{i}(f_{i}-\phi )$$

which is implemented as

(14) $$\begin{array}{cccc} & x_{i}(t+1)=x_{i}(t)+\eta \;x_{i}(t)(f_{i}-\phi ) & & \end{array}$$

where $\phi =\sum_{i}x_{i}f_{i}$ denotes the population’s average fitness and η is the learning rate. To enhance numerical stability and prevent oscillatory behavior, a momentum-based update rule is adopted:

(15) $$\begin{array}{c} \Delta x_{i}=\beta \Delta x_{i,prev}+(1-\beta )\eta_{eff}x_{i}(f_{i}-\phi )\\ \;\;\;\;\;\;\;\;\;\;\;\;\;\;\;\;x_{i}(t+1)=\mathrm{clip}(x_{i}(t)+\Delta x_{i},0.10,0.60) \end{array}$$

Implementation parameters:

- Momentum coefficient β=0.8.
- Weight bounds: [0.1, 0.6].
- Adaptive learning rate:

(16) $$\eta_{eff}=base\_lr\cdot (0.3+0.7\cdot deviation)$$

where *deviation* is the standard deviation of the fitness values. Nash convergence criterion: stability is assumed when deviation<0.02 for five consecutive epochs. This mechanism balances adaptation speed with convergence stability, preventing dominance collapse while preserving responsiveness to performance fluctuations.

Here, clip(*z*, *a*, *b*) = min(max(*z, a*), *b*) is applied element-wise and projects each updated weight back into the admissible interval [w_min, w_max] = [0.10, 0.60] (Equation (15)), preventing any single loss term from vanishing or dominating; the clipped weight vector is then renormalized so that it continues to satisfy the simplex constraint of Equation (9). In the adaptive learning rate of Equation (16), η_eff = base_lr·(0.3 + 0.7·deviation), the term “deviation” denotes the standard deviation of the three fitness values normalized to the unit interval [0, 1]; consequently, η_eff ranges from 0.3·base_lr when the fitness values are nearly equal (close to convergence) to 1.0·base_lr when they diverge maximally (early adaptation).

#### 2.5.4. EGT Configuration Variants

The replicator dynamics framework introduced above offers flexibility in tuning how aggressively the loss weights adapt during training as given in Table 1. Depending on the choice of learning rate and momentum parameters, the system can prioritize either convergence stability or rapid adaptation to changing segmentation conditions. To systematically analyze this trade-off, three distinct EGT configurations were designed and evaluated:

While the EGT Aggressive version is capable of quick adaptation owing to higher learning rates and regular updates, the EGT Boundary-Focus version ensures accurate contours by placing more emphasis on fitness associated with boundaries.

### 2.6. Ablation Study Design

Ablation studies were done to accurately measure the performance contribution of each of the architectural improvements as well as that of the proposed method for optimizing losses dynamically. Six model settings were tested, which are shown in Table 2.

This structured decomposition ensures that performance gains can be attributed to clearly isolated methodological components rather than confounding interactions.

### 2.7. Statistical Analysis

To determine whether observed performance differences were statistically significant, both parametric and non-parametric paired analyses were performed across the 191 independent test images. The statistical framework was designed in accordance with the reporting standards for deep learning models in medical imaging [37].

- **Wilcoxon Signed-Rank Test:** A non-parametric test for paired comparisons without assuming normal distribution.
- **Paired *t*-test:** Applied when normality assumptions were satisfied, enabling direct mean comparison.
- **Cohen’s d (Effect Size):** Quantifies the magnitude of performance differences:

(17) $$d=\frac{\mu_{1}-\mu_{2}}{s_{pooled}}$$

- **95% Confidence Intervals (CIs):** Estimated using the standard error method.

In the effect-size definition of Equation (17), μ_1_ and μ_2_ denote the mean scores of the two paired distributions being compared—typically the mean test-set metric of the candidate model and of the baseline, respectively—and s_pooled is the pooled standard deviation of the two samples; a positive d therefore indicates that the candidate model outperforms the baseline, with |d| quantifying the magnitude of the effect.

Statistical significance was defined at an alpha level of α=0.05. Although Bonferroni correction is available for multiple hypothesis testing, it was not applied in this study, as comparisons were restricted to pre-defined hypotheses across a fixed and independent test set. The use of paired statistical testing over 191 samples provides substantial statistical power, minimizing the likelihood that the reported improvements are attributable to random variation.

### 2.8. Evaluation Metrics

A reliable assessment of the accuracy of segmentation is necessary in order to provide correct decisions. Previous studies have established the fact that there is currently no metric that evaluates all possible aspects of segmentation and that class imbalance and non-uniform shapes are common in biomedical imaging [31,38]. As such, this study uses a series of different metrics that focus on overlap of regions, contour accuracy, confidence in models’ predictions, and pixel-level accuracy to obtain an overall score that represents the efficiency of segmentations of pediatric dental structures. The following metrics will be used [38,39,40]: The Dice coefficient measures the degree of overlap between predicted segmentation and the ground-truth segmentation and has been proven to be effective for evaluating the accuracy of medical image segmentation, especially due to being insensitive to class imbalance. It is calculated using

(18) $$\mathrm{Dice}=\frac{2|P\cap G|}{|P|+|G|}$$

where P is the predicted mask and G is the ground truth. Higher Dice values indicate better agreement between predictions and annotations. IoU, or the Jaccard index, evaluates the ratio of the intersection to the union of the predicted and ground-truth regions:

(19) $$\mathrm{IoU}=\frac{|P\cap G|}{|P\cup G|}$$

The IoU metric punishes both false positives and false negatives more strictly than the Dice metric does, making it an essential evaluation criterion for images in pediatrics where there is poor contrast at the borders. Pixel-wise accuracy evaluates the ratio of correct pixel predictions to the total number of pixels in the image:

(20) $$\mathrm{Accuracy}=\frac{TP+TN}{TP+TN+FP+FN}$$

This metric offers a global view of prediction correctness, although it can be biased in datasets with large background regions.

In the accuracy expression of Equation (20), TP, TN, FP and FN denote, respectively, the numbers of pixels correctly classified as teeth (true positives), correctly classified as background (true negatives), incorrectly classified as teeth (false positives) and incorrectly classified as background (false negatives), with the tooth region taken as the positive class.

The Hausdorff Distance at the 95th percentile (HD95) is a boundary-based metric that measures the maximum mismatch between the predicted and ground-truth contours, computed at the 95th percentile to reduce sensitivity to outliers [37]. It is defined as

(21) $$HD95=max\left(h_{95}(P,G),h_{95}(G,P)\right)$$

where $h_{95}(A,B)={\mathrm{percentile}}_{95}{\left({min}_{b\in B}∥a-b∥\right)}_{a\in A}$. Lower HD95 values indicate closer agreement between predicted and true boundaries.

The Average Surface Distance (ASD) quantifies the mean distance between the surface of the predicted mask and that of the ground truth, providing a symmetric measure of boundary deviation [38]:

(22) $$ASD=\frac{1}{2}\left(\frac{\sum_{p\in \partial P}d(p,\partial G)}{|\partial P|}+\frac{\sum_{g\in \partial G}d(g,\partial P)}{|\partial G|}\right)$$

where ∂P and ∂G denote the boundary point sets of the predicted and ground-truth masks, respectively, and d(⋅,⋅) is the minimum Euclidean distance to the opposing surface. Lower ASD values reflect more precise boundary localization. The Boundary Dice (BDice) score evaluates segmentation quality specifically along object boundaries by restricting the Dice computation to a narrow band τ around the contour

(23) $$BDice=\frac{2|\partial_{\tau }P\cap \partial_{\tau }G|}{|\partial_{\tau }P|+|\partial_{\tau }G|}$$

where $\partial_{\tau }P$ and $\partial_{\tau }G$ represent the sets of predicted and ground-truth boundary pixels within a tolerance band of width τ. In this study, τ was set to 2 pixels, consistent with prior work in medical image segmentation [41]. Higher Boundary Dice values indicate more accurate delineation of tooth contours.

## 3. Experimental Results

All experiments were carried out using a high-performance workstation optimized for pediatric dental segmentation using deep learning techniques. The system used an NVIDIA RTX 4090 GPU with 24 GB VRAM, 64 GB RAM, and an Intel Core i9 multi-core CPU. The software configuration was set up under the Windows 11 Pro operating system, with the use of Python 3.7.4 and TensorFlow 2.10.0, alongside additional libraries such as OpenCV 4.11.0, scikit-learn 1.6.1, and Matplotlib 3.10.1, CUDA acceleration enabled the effective training process on a dataset containing 1269 pediatric panoramic radiograph images.

### 3.1. Training and Data Pipeline

An organized ablation study was conducted using six different model setups to effectively attribute the impact of each architectural improvement, hybrid loss function design, and EGT-inspired optimization to the Baseline U-Net model. The above procedure will ensure that the evaluation of the performance comparison for all ablation experiments will be scientifically robust, impartial, and reproducible. A two-step evaluation methodology was used:

(1) An 85%/15% train/test split per patient, ensuring that there are no overlapping subjects between the two sets.
(2) Five-fold cross-validation for the training set. The entire preprocessing and augmentation procedure is listed in Table 3.

This pipeline ensures both anatomical fidelity and generalization robustness under varying illumination and structural conditions.

### 3.2. Quantitative Performance Analysis

**Figure 3:**
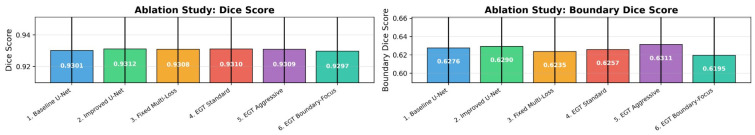
Ablation study results illustrating Dice and Boundary Dice performance across model configurations.

Table 4 presents the performance evaluation for the independent testing dataset (*n* = 191). All the models achieved a Dice score higher than 0.93, which means that the problem of segmentation is properly structured and that the baseline performance is relatively strong. Figure 3 shows the ablation experiment results.

**Figure 4:**
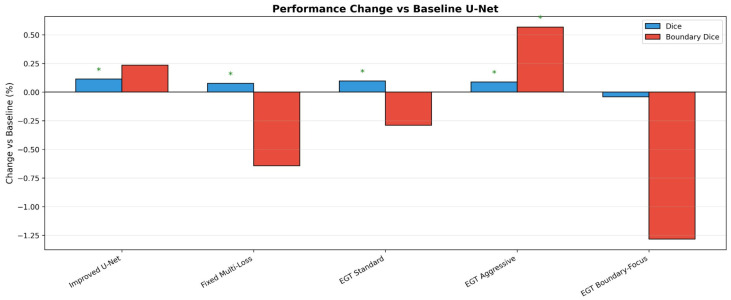
Relative performance change (%) compared to the Baseline U-Net. Blue bars represent Dice score variation, while red bars indicate Boundary Dice variation. The green asterisk (*) denotes statistically significant differences according to the Wilcoxon signed-rank test (*p* < 0.05).

Comparison with the Baseline U-Net on statistical tests for all models was conducted to evaluate their overall performance. The results are presented in Table 5 below. The analysis of statistical results shows that all models but one, namely, EGT Boundary-Focus, were able to statistically outperform the baseline model on Dice scores. Even though the gains in terms of percentage are not large (+0.076% to +0.113%), statistical significance at *p* < 0.05 level and small effect size, i.e., Cohen’s *d* = 0.070–0.100, are confirmed by the Wilcoxon signed-rank test. Only the EGT Boundary-Focus model demonstrated no statistical significance (*p*-value = 0.7697). As for the Boundary Dice metric (see Table 6 below), a more discriminatory result can be found. EGT Aggressive appears to be the only model that achieved a statistically significant positive effect in comparison with the Baseline U-Net (Wilcoxon *p*-value = 0.0127, Cohen’s *d* = 0.184; thus, moderate effect size). However, Fixed Multi-Loss configuration showed statistically significant negative effect on Boundary Dice score (*p*-value = 0.0208, *d*= −0.185), demonstrating that the static configuration leads to possible optimization conflicts. Moreover, EGT Boundary-Focus model demonstrates statistically significant decrease in its Boundary Dice performance (*p*-value < 0.001, *d* = −0.344). An important conclusion made based on the analysis above is that EGT Aggressive is the only model that demonstrates statistically significant positive performance change on both considered metrics—Dice and Boundary Dice: Dice (*p*-value = 0.0144, *d* = 0.070; small), Boundary Dice (*p*-value = 0.0127, *d* = 0.184; small-to-moderate). It should be noted that all other tested strategies appear to be worse compared to Baseline and/or EGT Aggressive. Relative performance change (%) compared to the Baseline U-Net is shown in Figure 4.

The results from the statistical comparison between other metrics are presented in Table 7. Effect sizes (Cohen’s d) are small in this case; however, this trend corresponds to the expectations for high-baseline segmentation tasks with performance saturation. In such cases, statistically significant gains in performance between pairs of samples show the effectiveness of introduced modifications. One peculiar result is the statistically significant decrease in the Boundary Dice for the Fixed Multi-Loss setting. This observation shows that static weighting of multiple objectives can lead to an optimization conflict between edge-aware losses and other region-related metrics. Moreover, the insufficient performance gain of EGT Boundary-Focus demonstrates that the excessive weight of a particular metric used in the evolution target function can result in local minima or imbalance between global and local optimization goals. On the other hand, the consistent increase in Recall values in all multi-loss settings (*p* < 0.05) proves that the use of Focal Tversky and Boundary losses leads to a better reduction in false negatives, which is crucial from a clinical perspective since false negatives (missed teeth in this case) are typically more important than false positives.

### 3.3. EGT Dynamic Weight Evolution

Under EGT-based settings, the loss-weighting coefficients undergo an adaptive evolution throughout the training process according to replicator dynamics. In this part, we provide a comprehensive study of the evolutionary dynamics under the three suggested EGT frameworks, focusing mainly on their convergence characteristics.

#### 3.3.1. In-Depth Analysis of EGT Aggressive

The EGT Aggressive variant exhibited the most pronounced adaptive behavior and yielded the strongest performance gains. Over 48 evolutionary generations, the strategy vector shifted from a near-balanced initialization to a boundary-dominant equilibrium (Table 8).

The above evolution process clearly illustrates the transformation of the model from a perfectly balanced scenario into one where boundaries played a dominant role. The weighting for the Dice loss was reduced by 71% on the other hand, and the weighting for the Boundary loss was increased by over two times. This indicates that the model learned globally first before focusing on the boundaries.

- Epochs 5 to 20 (beginning phase after warmup): An abrupt increment in the Boundary Weight becomes evident, in tandem with the beginning drop in the Dice weight.
- Epochs 20 to 60 (middle phase): Linear evolution continues, with the Boundary Weight crossing the threshold value of 0.50.
- Epochs 60 to 100 (ending phase): There occurs a phase of stability, with the Dice weight getting close to the lower limit of clipping (approx. 0.10).

**Figure 5:**
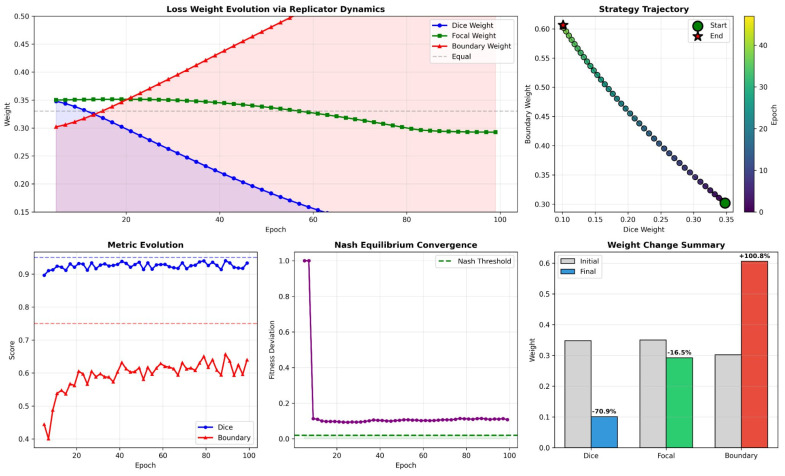
EGT Aggressive configuration: loss weight evolution (**top-left**), strategy trajectory (**top-right**), metric evolution (**bottom-left**), Nash convergence (**bottom-center**), and weight change summary (**bottom-right**).

As seen in Figure 5, bottom right, the strategy evolution graph shows the movement of weights on the Dice Weight vs. Boundary Weight graph. The movement of weights is continuous and monotonically increasing from the green initial state of (0.35, 0.30) to the red final state of (0.10, 0.61). Therefore, this demonstrates the absence of any oscillations when using momentum-based replicator dynamics.

#### 3.3.2. EGT Standard Analysis

Among the three configurations, EGT Standard adopted a more conservative evolutionary pattern, reflecting a cautious balance between adaptation speed and stability. The model was trained for 31 generations with moderate weight changes, rebalancing the optimization pressure gradually rather than abruptly. In particular, the Dice weight was reduced most significantly, from 0.350 to 0.242, corresponding to a decrease of 30.8%. The Focal Weight was relatively stable throughout training, only changing slightly from 0.350 to 0.346 (−1.1%). In contrast, Boundary Weight exhibited a steady increase from 0.300 to 0.412, a 37.3% increase, suggesting that this setting allowed the model to gradually realize the boundary delineation as the most important optimization goal.

**Figure 6:**
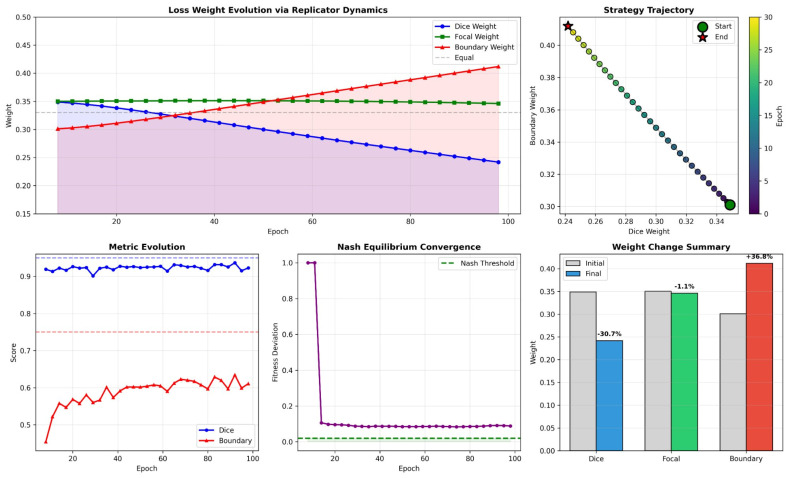
EGT Standard configuration: loss weight evolution and dynamic behavior analysis. A conservative evolutionary pattern and slower convergence are observed compared to the aggressive variant.

The use of a smaller learning rate (η = 0.15) and infrequent weight updates (every three epochs) limited the pace and degree of evolution, as shown in Figure 6. As such, even though the enhancement of boundaries was increased, it failed to reach the level of dominance necessary to achieve any significant gains in Boundary Dice score (*p* > 0.05). This outcome demonstrates the critical compromise that must be struck, as insufficient evolutionary pressure can ensure stability while being unable to overcome optimization plateaus.

#### 3.3.3. The Paradox of EGT Boundary-Focus

Despite assigning a heavily skewed boundary importance within the fitness formulation ($w_{fitness}=0.8$), the EGT Boundary-Focus configuration yielded unexpectedly weaker boundary-specific performance. The observed weight evolution was modest:

- Boundary Weight: +31% increase, converging at 0.393.

This paradox suggests that premature over-prioritization of boundary fidelity may suppress the foundational learning of global structure (Dice) and hard-sample sensitivity (Focal). Without sufficiently stable region-level segmentation, excessive edge emphasis can amplify noise and local inconsistencies rather than refine meaningful contours. This outcome underscores a central principle of multi-objective optimization: balanced evolutionary adaptation outperforms singular objective amplification. Rather than statically privileging one metric, the EGT framework achieves superior performance when fitness-based adaptation dynamically responds to shifting bottlenecks during training.

**Figure 7:**
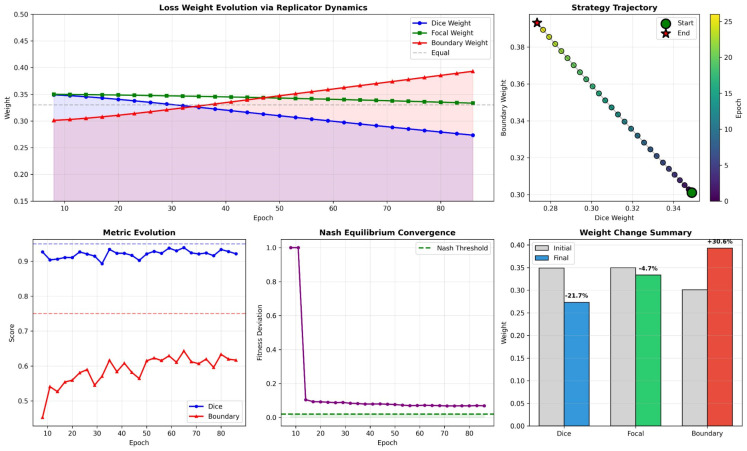
EGT Boundary-Focus configuration illustrating an unexpectedly conservative evolutionary trajectory and reduced performance, indicating that excessive emphasis on boundary fitness may hinder balanced multi-objective optimization.

As illustrated in Figure 7, the EGT Boundary-Focus model demonstrates relatively conservative evolution, along with reduced segmentation capacity. The evolution path for the algorithm suggests that the boundary-centric goal rapidly gains dominance, while the influence of the Dice and Focal Tversky losses steadily decreases. Such an imbalance causes poor convergence behavior, similar to the Nash equilibrium game, where the system achieves stability through an approach that favors boundaries.

### 3.4. Model Training Duration

The training durations of the models were evaluated in terms of computational cost. All experiments were conducted on the same hardware configuration. Comparison of model training times is given in Table 9.

The training time study found that the Baseline U-Net achieved the fastest training time (13.4 min) and therefore provided a benchmark for computation. Incorporating the Attention and SE blocks into the architecture increased training time by around 51%, due to the higher complexity of the model. The computational expense incurred as a result of EGT optimization was fairly low, at around 13%, compared to the Fixed Multi-Loss design. It is interesting to observe that EGT Aggressive and EGT Standard models had similar training times since the benefits of the increased adaptability outweighed those of the shorter number of iterations needed for convergence. From a computational standpoint, the increase in training time of around 55% in the EGT approach is still reasonable when the statistically significant improvement in results is taken into account. Crucially, the inference time was constant across all designs, amounting to roughly 5.2 ms per image.

The EGT controller optimizes only a three-dimensional weight vector at a few-epoch cadence and modifies no network parameters, so its arithmetic cost is negligible relative to a forward/backward pass; the only measurable overhead is the periodic validation pass used to compute fitness. As reported in Table 9, EGT Standard trains in 20.9 min versus 13.4 min for the Baseline U-Net (1.56×) and the Fixed Multi-Loss model (18.3 min), and it incurs zero additional inference cost because the learned weights are frozen at deployment.

### 3.5. Comprehensive Metric Comparison

**Figure 8:**
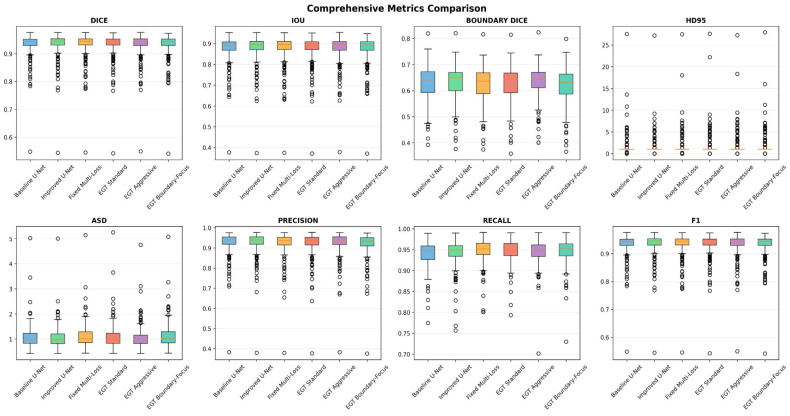
Comprehensive metric comparison: box-plot distributions of Dice, IoU, Boundary Dice, HD95, ASD, Precision, Recall, and F1 scores for all models.

To provide a more holistic picture of the model performance than point estimates, the distributional properties of all evaluation metrics over the six architectures are provided in box-plots in Figure 8. This visualization shows the central tendency of each metric through the median and the spread of predictions through the interquartile range (IQR) to provide a more nuanced picture of model consistency and robustness. In addition, potential outliers are made visible, highlighting cases where individual models failed with particularly challenging segmentation scenarios.

The box-plot analysis indicates a number of key trends across the models assessed. First, the high correlation of Dice and IoU distributions for all architectures means the basic segmentation objective is achieved successfully and there is no substantial difference between overlap-based metrics. However, looking at Boundary Dice, there emerge more significant differences between models. EGT Aggressive gets the highest median score across all configurations, highlighting the benefit of aggressive adaptive weighting for boundary-sensitive tasks. The presence of outliers in multiple model distributions in HD95 points to anatomically difficult cases, where precise boundary localization remains challenging, likely related to images with high structural overlap or advanced mixed dentition. Finally, on Recall, Fixed Multi-Loss and EGT-based models consistently outperform the rest of the strategies. This indicates that hybrid and adaptive loss formulations are especially good at minimizing missed tooth regions.

**Figure 9:**
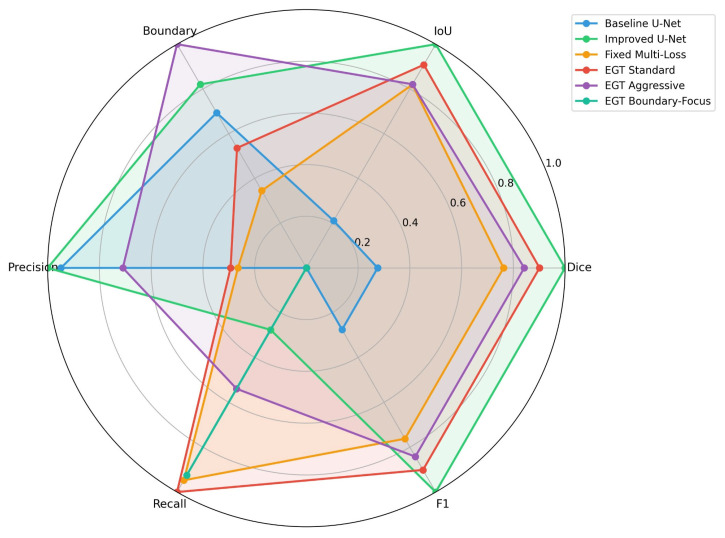
Multi-metric radar chart: Normalized performance profiles of all models. Each axis represents a distinct evaluation metric.

The radar chart illustrates the relative performance of each model across six core metrics as shown in Figure 9. EGT Aggressive (purple) and Improved U-Net (green) cover the largest areas, indicating stronger overall performance profiles. The superiority of EGT Aggressive in the Boundary metric is particularly evident in the radar visualization.

### 3.6. Complexity Analysis

**Figure 10:**
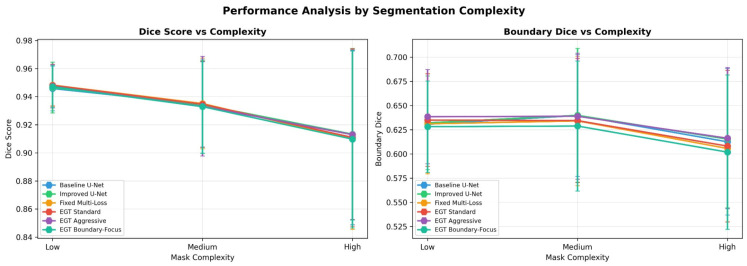
Performance analysis according to segmentation complexity: Dice (**left**) and Boundary Dice (**right**) metrics.

Model performance was further analyzed with respect to segmentation complexity, quantified using the perimeter-to-area ratio of the ground-truth mask for each image. Based on this ratio, images were categorized into three tertiles representing low, medium, and high complexity levels. Figure 10 presents the corresponding performance variation across these complexity groups using Dice (left) and Boundary Dice (right) metrics.

As mask complexity increases from low to high, a gradual decline in Dice scores is observed across all models, confirming the expected trend that structurally intricate regions pose greater segmentation challenges. Notably, while all architectures perform at a comparable level under low-complexity conditions, performance differences become increasingly pronounced as complexity rises, revealing the discriminative power of high-complexity cases in distinguishing between model designs. This pattern is particularly evident in Boundary Dice scores, where EGT Aggressive demonstrates a clear advantage over competing configurations, especially in the high-complexity tertile. This finding suggests that the adaptive loss-weighting mechanism inherent to the EGT framework confers the greatest benefit precisely in the most challenging segmentation scenarios, where rigid, fixed-weight approaches tend to fall short.

**Figure 11:**
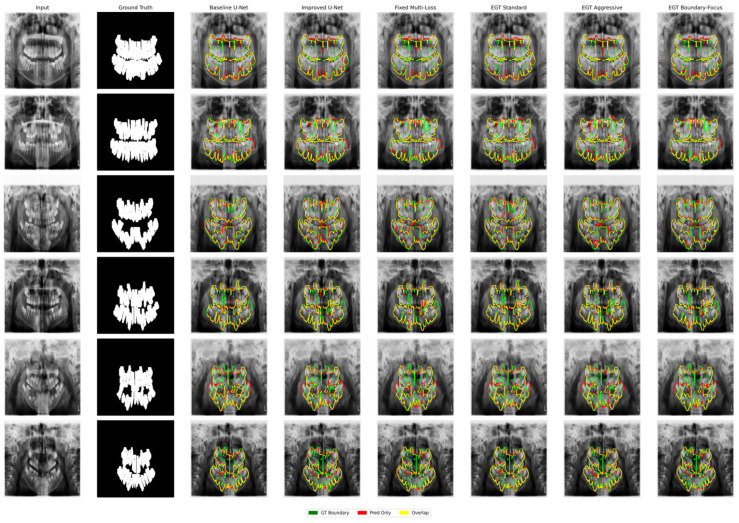
Qualitative comparison: segmentation results across different complexity levels. Green: ground-truth boundary, Red: prediction only (FP), Yellow: overlap.

Figure 11 presents a visual comparison of segmentation results from all models on selected test images representing different complexity levels. In the boundary overlay visualization, green denotes the ground-truth boundary, red indicates model-only predictions (false positives), and yellow represents overlapping regions.

From visual examination, all models can be considered good representations of the tooth structure. However, qualitative differences were observed between various architectures. The Baseline U-Net showed a larger number of false positives, mainly in the interproximal area, while the EGT Aggressive configuration produced cleaner boundaries and segmentation of the root and occlusal parts of teeth. In more difficult cases, such as when there was an overlap of several teeth in one image, the EGT-based approaches performed better with regard to segmentation artifacts. For better understanding of how the model works during the inference stage, the Gradient-weighted Class Activation Map (Grad-CAM) method was used to determine the important parts of the images that played a crucial role in segmentation.

**Figure 12:**
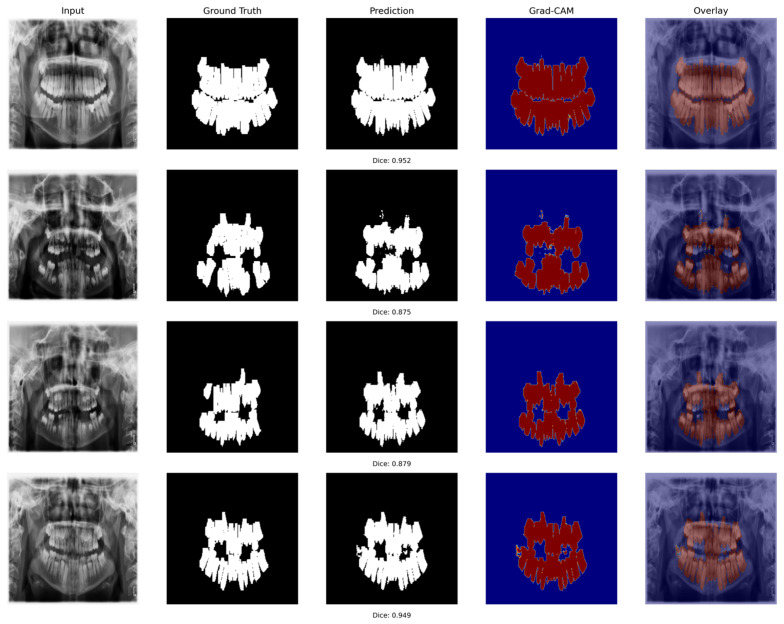
Grad-CAM analysis: Baseline U-Net. Activations are concentrated in tooth regions; however, spillover beyond boundary regions is observed.

Based on the Grad-CAM analysis, it can be seen that there are differences in attention patterns depending on the model settings. While the Baseline U-Net (in Figure 12) shows less focal activations, highlighting parts of the image that at times go into the background, the EGT Aggressive configuration has more focal activations that correlate well with the anatomical structure of the tooth region being segmented. As for the Improved U-Net (Attention + SE), it shows a certain focus but its activations are not as sharply defined as those in the case of EGT Aggressive. All of this suggests that the EGT boundary-aware loss optimization is capable of focusing network attention around the boundaries between teeth and their surrounding regions.

On a practical level, it could be argued that the proposed framework of utilizing EGT-UNet in dental imaging is useful for clinicians dealing with pediatric patients who have mixed dentitions and low-contrast interface between teeth due to their different stages of eruption. In general, while the differences between the Dice scores are relatively small, improving the boundary detection capabilities can help to make orthodontic measurements and assessments of root morphology more accurate and reliable.

### 3.7. Comparison with Other Segmentation Architectures

**Figure 13:**
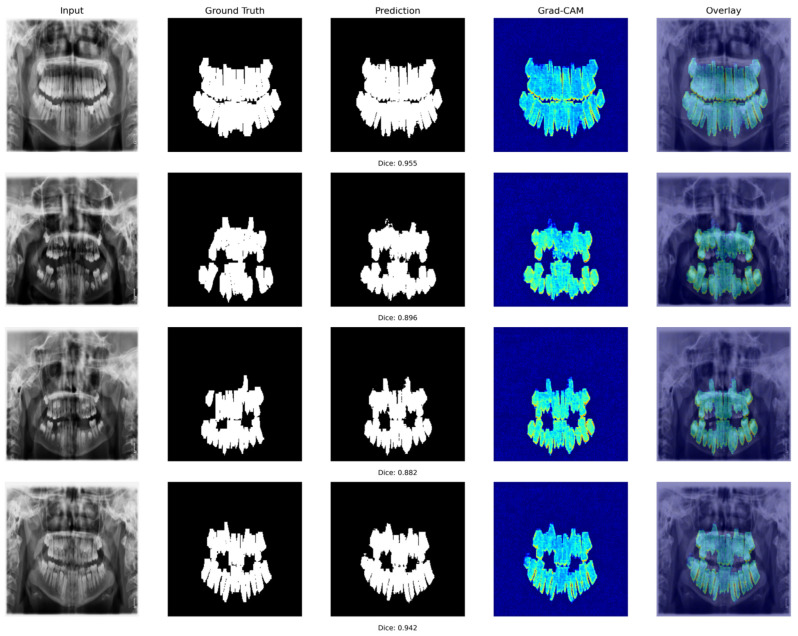
Grad-CAM analysis: EGT Aggressive. More focused and homogeneous activation distribution, with attention concentrated closer to boundary regions.

To address the cross-architecture comparison requested in review, the proposed EGT-UNet is benchmarked against representative models from the major architecture families under identical conditions—the same dataset, the same patient-level train/test split, and identical preprocessing and evaluation protocols: a CNN Baseline (U-Net), a multi-scale CNN (UNet 3+), and a Transformer-based model (Swin-UNet). The aggregated results are reported in Table 10. Grad-CAM analysis of EGT Aggressive is given in Figure 13.

**Figure 14:**
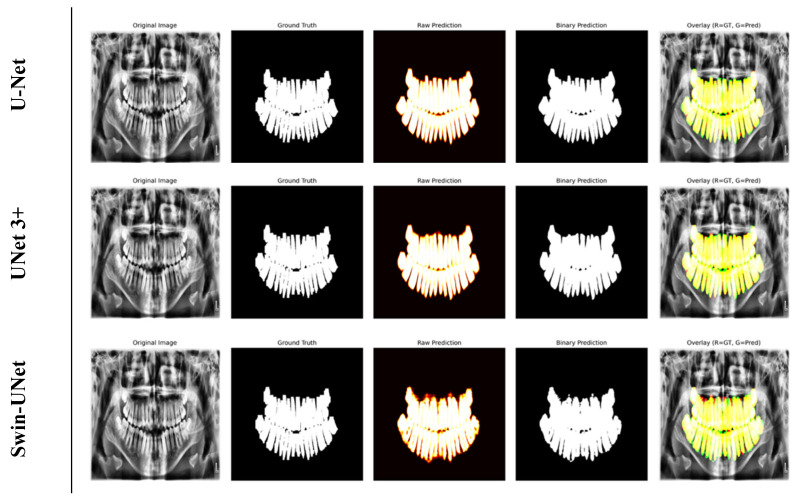
Qualitative visualization of used models.

**Figure 15:**
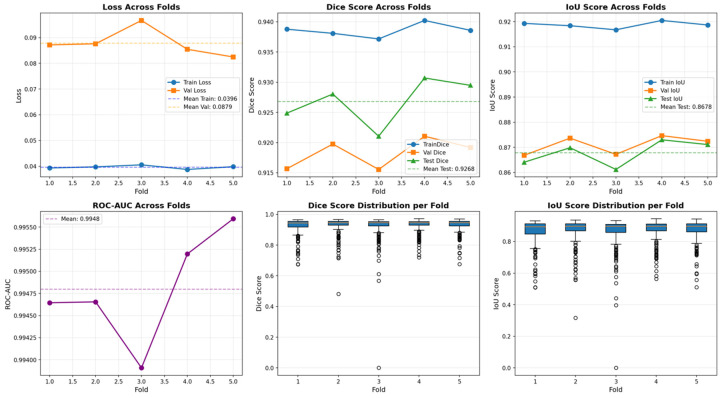
Five-fold cross-validation curves and per-fold Dice/IoU distributions of the U-Net Baseline.

**Figure 16:**
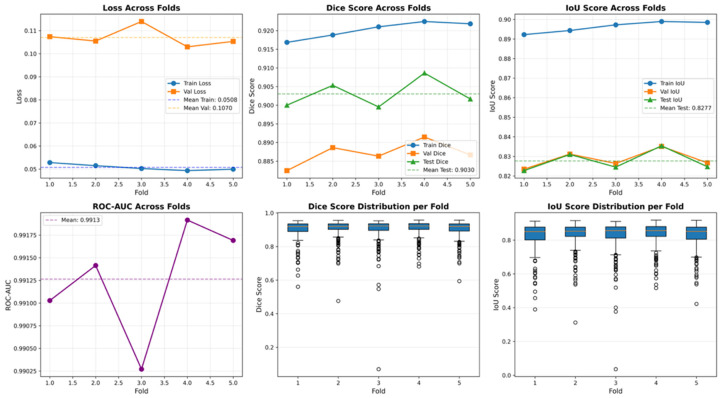
Five-fold cross-validation curves and per-fold Dice/IoU distributions of the Swin-UNet model.

**Figure 17:**
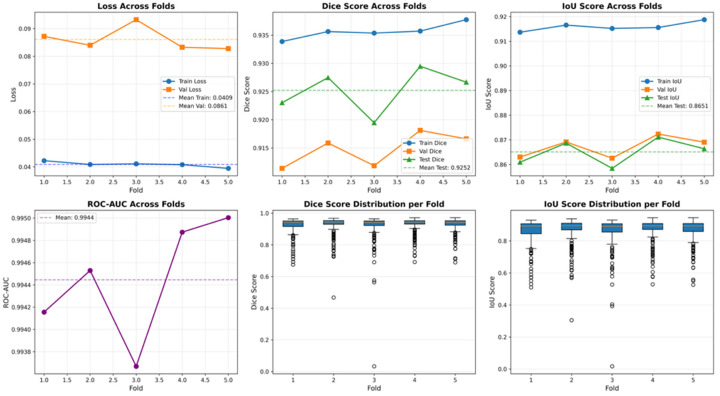
Five-fold cross-validation curves and per-fold Dice/IoU distributions of the UNet 3+ model.

EGT-UNet attains the highest Dice and IoU and, in particular, outperforms the Transformer-based Swin-UNet by a clear margin (Dice 0.931 vs. 0.907). This is consistent with the well-documented tendency of pure-Transformer segmentation models to underperform CNNs in limited-data regimes without large-scale pretraining, which is precisely the pediatric setting considered here. Because the proposed EGT loss-weighting scheme is backbone-agnostic, the relevant comparison axes are loss optimization strategies on a common backbone (the six-model ablation) and architecture families; self-configuring pipelines such as nnU-Net and additional dental-specific Transformers (e.g., TransUNet) are left to future work, as their automated design would confound the isolated effect of the loss-weighting mechanism studied here. Qualitative visualization of used models are shown in Figure 14. The five-fold cross-validation behavior of each comparison model is shown in Figure 15, Figure 16 and Figure 17.

### 3.8. Comparison with State-of-the-Art Methods

Comparing the proposed EGT-UNet with previous works in dental imaging as shown in Table 11, several notable differences emerge, particularly in terms of patient population, dataset size, and the inherent difficulty of the segmentation tasks. The majority of prior studies were conducted on adult patients using panoramic radiographs or three-dimensional CBCT scans, where tooth morphology is well preserved and datasets are relatively straightforward to annotate. Pediatric panoramic radiography, by contrast, presents a considerably more challenging environment for segmentation algorithms. This heightened complexity stems from a combination of factors unique to this imaging context: the simultaneous presence of mixed dentition alongside erupting permanent teeth, the progressive resorption of primary roots, and the frequent overlap between distinct anatomical structures. Further complicating matters are inconsistent tooth mineralization patterns, irregular interdental spacing, motion artifacts inherent to pediatric imaging, and the critically limited availability of annotated datasets specifically tailored to this population. Taken together, these characteristics make pediatric panoramic radiograph segmentation a distinctly demanding problem that existing methods have largely left unaddressed.

The poor performance of segmentation algorithms when working with pediatric patients was proven by works like [26,29]. In addition to that, there is also another difference that can be observed—many previous studies are focused on datasets extracted from adult patients, suggesting that these models are not fully adapted to segment pediatric dental data. In this regard, EGT-UNet designed by our group reached Dice = 0.931 and IoU = 0.873 on pediatric radiography datasets, which is equal or better than adult-based segmentation models, and much better than any other Transformer or generic model tested on pediatric data.

## 4. Discussion

This work presents EGT-UNet, an innovative pediatric panoramic tooth segmentation solution incorporating architectural innovations as well as a novel dynamic loss-weighting mechanism based on Evolutionary Game Theory (EGT). A comprehensive ablation study comparing six model configurations highlights the importance of both of these contributions. While all six configurations outperform the baseline in terms of Dice scores on the hold-out test set (greater than 0.93 Dice for all configurations), the difference between model performances is more pronounced in metrics sensitive to tooth boundaries. This suggests that the main obstacle to achieving accurate segmentation results on this problem remains accurate tooth boundary definition, rather than simply maximizing the area of overlap between segmented teeth and target regions. Of all comparisons performed in our ablation study, the comparison between the Fixed Multi-Loss and EGT-based configurations was the most telling, since both configurations use the same three-component loss function (Dice, Focal Tversky, and Boundary) but use different loss-weighting strategies. While the fixed weighting scheme used in the former configuration led to a significant decrease in boundary-related metric (−0.64%, *p* < 0.05), the evolutionarily optimized loss weight configuration (EGT Aggressive) was the only one to yield a significant increase in both Dice and Boundary Dice values compared to the baseline (Wilcoxon signed-rank test, *p* < 0.05). It is evident from Table 8 and Figure 5 that the evolutionary trajectory of the EGT Aggressive configuration involved increasing the contribution of the Boundary loss component from 0.300 to 0.606 (increase by 102%) and decreasing the weight of the Dice loss (from 0.350 to 0.101; decrease by 71.1%) as the process of optimizing the global region of the tooth became saturated. Thus, the optimizer was able to dynamically adapt the weight distribution, putting a stronger focus on Boundary loss as opposed to the fixed ratio. As illustrated in Figure 5, momentum-stabilized replicator dynamics led to monotonic evolution of the strategy vector toward an optimal point.

Comparison of EGT configurations highlights the need for careful selection of hyperparameters when employing evolutionary algorithms in machine learning problems. While EGT Standard configuration exhibited consistent improvements in performance in terms of direction but failed to show statistical significance of gains, the performance of the explicitly Boundary-Focused variant, on the contrary, turned out worse in some metrics. These observations highlight the importance of allowing sufficient freedom of movement for the optimizer in the evolutionary strategy space. Finally, improvement of architecture was demonstrated by moving from Baseline U-Net to Improved U-Net, with the latter providing better HD95 metric (1.61 ± 2.47, minimum among all models) due to the introduction of SE blocks, Attention Gates, and ASPP block. Indeed, qualitative analysis of gradient activations (Grad-CAM) provided in Figure 12 and Figure 13 confirmed that EGT Aggressive configuration produces sharper and more localized activation patterns that are highly correlated with true tooth boundary localization. Finally, Figure 10 shows that complexity stratification analysis indicates the greatest advantages of EGT-based adaptation on high-complexity cases (overlapping teeth, incomplete eruptions, and resorbing primary teeth), corresponding to the highest workload for the clinicians. In relation to the state-of-the-art in the field of dental segmentation (Table 11), our model demonstrates competitive performance on an exclusive pediatric sample of images, making its task significantly harder than in previous studies focusing on adults. In most prior works, high performance (usually close to 100% Dice score or IoU) was reported for segmentation of teeth in adult panoramic radiographs or CT images, where tooth structure is much less variable and image contrast is higher. For pediatric datasets, Rubiu et al. obtained a DSC score of 0.87 using Mask R-CNN on an adult–child mixed dataset. Asci et al., working on caries detection in children, scored an F1 of 86.75%. Compared to these results, our model obtains a Dice of 0.931 on a purely pediatric set of panoramic images. With 1269 annotated panoramic images publicly available, this dataset makes a long-desired step toward solving the shortage of publicly available pediatric medical AI datasets. From a clinical perspective, while the absolute improvements in Dice score are relatively modest, the improved boundary accuracy can be considered valuable in orthodontic applications, where accurate boundaries influence various measures of tooth width and angle, arch spacing calculations, etc. Consistent statistical significance of gains in paired comparisons of metrics suggests that these gains have clinical relevance, especially in the context of numerous teeth contained in each panoramic image. Some limitations, however, remain: single-center origin of the dataset can introduce bias in data distribution, multi-center validation is needed. Effect sizes observed in this study are relatively small because of high baseline values. Finally, the choice of hyperparameters for the evolutionary algorithm was made on an empirical basis. For future research, multi-center validation, development of a system capable of assigning unique identifiers to each tooth, application of pretraining techniques with semi-supervised learning to minimize annotation needs, and use of other boundary-based losses in an EGT scheme are proposed.

To the best of our knowledge, no public pixel-level pediatric panoramic segmentation dataset currently exists; the curated dataset released with this work is, to our knowledge, the first of its kind, which is why all images originate from a single institution. We therefore state explicitly that external validity across scanners, acquisition protocols, and populations has not yet been established, and we identify multi-center and cross-device external validation as the primary direction of future work. The EGT mechanism adds only about 3% to training time over the Fixed Multi-Loss model and incurs zero additional inference cost, so its cost–benefit trade-off is favorable even though the absolute Dice improvement over the strong improved baseline is small; we refrain from claiming a definitive clinical effect and note that this requires prospective, multi-reader evaluation. The boundary-level improvement of the EGT Aggressive configuration (+0.567%, *p* = 0.0127) is of a magnitude comparable to inter-expert annotation variability, so we interpret it as improved contour consistency rather than a decisive clinical advantage, and we acknowledge that some configurations (e.g., Boundary-Focus) degrade boundary performance, reflecting a genuine trade-off. Qualitative examples of successful and failed segmentations in complex mixed dentition cases are provided to support interpretability. Recent systematic reviews of AI-based dental and CBCT tooth segmentation consistently highlight limited and heterogeneous datasets, non-standardized evaluation metrics, and barriers to clinical translation—challenges that directly motivate both the curated pediatric dataset and the standardized, multi-metric evaluation protocol adopted in this study. Beyond comparisons with individual pediatric segmentation studies, it is useful to situate the present work within the broader landscape of AI-based dental image segmentation. The recent systematic review by Tarce et al. [44], which surveyed the application of artificial intelligence for tooth segmentation in CBCT images, provides a comprehensive synthesis of the deep learning architectures (predominantly U-Net-based and, increasingly, Transformer-based models), annotation strategies, and evaluation metrics used across the field, together with the recurring barriers to clinical translation. Several of the trends identified in that review—reliance on small, single-center, and often proprietary datasets, heterogeneous and non-standardized metric reporting, and limited external validation—closely mirror the challenges encountered in 2D panoramic segmentation and are directly addressed in our study through the release of a large, publicly available pediatric panoramic dataset and a standardized, multi-metric evaluation protocol. Meaningful differences nonetheless remain between 2D panoramic and 3D CBCT segmentation pipelines: CBCT-based approaches exploit volumetric information that facilitates separation of anatomically overlapping structures and, in several of the architectures reviewed by Tarce et al. [44], supports instance-level tooth identification, whereas panoramic radiographs remain the most widely available and cost-effective imaging modality in routine pediatric dental practice despite the inherent structural superimposition and lower spatial resolution that they present. Our EGT-based dynamic loss-weighting mechanism was designed specifically to mitigate the boundary-related difficulties arising from this 2D superimposition, and the gains observed in boundary-sensitive metrics suggest that game-theoretic loss balancing may offer a complementary strategy to the architectural innovations, such as attention mechanisms and graph-based anatomical modeling, that dominate the CBCT segmentation literature. We therefore regard the proposed framework not as a substitute for 3D approaches but as a modality-specific contribution that extends current AI-driven dental segmentation research to the pediatric panoramic domain, where, as highlighted by recent reviews, dedicated architectures and dedicated datasets remain scarce.

## 5. Conclusions

Our work introduces a novel EGT-UNet architecture for pediatric tooth segmentation from panoramic radiographs, which uses an advanced U-Net structure and integrates a dynamic learning rate weighting mechanism based on Evolutionary Game Theory (EGT). Specifically, our major contribution is the derivation of multi-loss optimization balancing as an evolutionary process driven by replicator dynamics using task-specific fitness functions for developing a theoretically justifiable yet transparent method compared to manually set learning rate coefficients. Experiments were carried out on our own developed dataset with 1269 panoramic images applying a strict patient-wise data splitting and five-fold cross-validation scheme. Our EGT Aggressive model scored a Dice of 0.931 ± 0.044 and a Boundary Dice of 0.631 ± 0.064 being the only version where both metrics were significantly increased compared to the baseline (Wilcoxon, *p* < 0.05). According to the ablation analysis, Fixed Multi-Loss balancing can lead to optimization problems, while the EGT balancing successfully re-balances learning rates towards under-represented loss functions. In addition, stratified results revealed the largest gains in complexity of segmentation tasks among pediatric patients with mixed dentition and overlapping tooth structures. This newly developed pediatric dental database along with an innovative multi-loss optimization algorithm provide grounds for further developments in the field of instance-level segmentation and multi-center validation.

## Figures and Tables

**Table 1:** Parameter details.

Parameter	EGT Standard	EGT Aggressive	EGT Boundary-Focus
Base Learning Rate	0.15	0.30	0.15
Update Frequency	3 epochs	2 epochs	3 epochs
Warmup Period	8 epochs	5 epochs	8 epochs
Momentum (β)	0.8	0.8	0.8
Fitness Boundary Weight	0.6	0.6	0.8
Initial $\left[D,F,B\right]$	[0.35, 0.35, 0.30]	[0.35, 0.35, 0.30]	[0.35, 0.35, 0.30]
Final $\left[D,F,B\right]$	[0.24, 0.35, 0.41]	[0.1, 0.29, 0.61]	[0.27, 0.33, 0.39]

**Table 2:** Model configurations for the ablation study.

No	Model	Architecture	Loss Function	Weighting Strategy
1	U-Net (Baseline)	Standard U-Net	Dice only	Fixed (1.0)
2	Improved U-Net	Attention + SE + ASPP	Dice only	Fixed (1.0)
3	Fixed Multi-Loss	Attention + SE + ASPP	Dice + Focal + Boundary	Fixed [0.35, 0.35, 0.30]
4	EGT Standard	Attention + SE + ASPP	Dice + Focal + Boundary	Dynamic (lr = 0.15)
5	EGT Aggressive	Attention + SE + ASPP	Dice + Focal + Boundary	Dynamic (lr = 0.30)
6	EGT Boundary-Focus	Attention + SE + ASPP	Dice + Focal + Boundary	Dynamic (boundary-weighted)

**Table 3:** Data preprocessing and augmentation pipeline.

Phase	Operation	Implementation Detail	Objective
Loading	Image Reading	png, jpg, tif, bmp	Mask–image alignment
Normalizing	Resizing	256×256	Input standardization
	Scaling	image/255.0	Pixel range $\left[0,1\right]$
	Thresholding	mask > 0.5 → 1.0	Ground-truth binarization
Enhancement	CLAHE	clip_limit = 0.01	Local contrast enhancement
	Gaussian Filter	σ=0.5	Noise suppression
Augmentation	Flipping and Rotation	p=0.5, ±90°	Spatial invariance
	Brightness	p=0.3, [0.8, 1.2]	Illumination robustness
Post-Process	Morphological	3×3 elliptic kernel	Artifact removal
	Watershed	min_distance = 10	Separation of adjacent teeth

**Table 4:** Comprehensive model performance comparison (*n* = 191).

Model	Dice	IoU	Boundary Dice	HD95	Precision	Recall	F1 Score
U-Net (Baseline)	0.930 ± 0.044	0.872 ± 0.066	0.628 ± 0.065	1.64 ± 2.60	0.923 ± 0.061	0.940 ± 0.030	0.930 ± 0.044
Improved U-Net	0.931 ± 0.044	0.874 ± 0.067	0.629 ± 0.065	1.61 ± 2.47	0.923 ± 0.062	0.942 ± 0.033	0.931 ± 0.044
Fixed Multi-Loss	0.931 ± 0.046	0.873 ± 0.069	0.624 ± 0.067	1.71 ± 2.76	0.918 ± 0.065	0.946 ± 0.030	0.931 ± 0.046
EGT Standard	0.931 ± 0.045	0.874 ± 0.067	0.626 ± 0.066	1.74 ± 2.90	0.919 ± 0.065	0.947 ± 0.027	0.931 ± 0.045
**EGT Aggressive**	**0.931 ± 0.044**	**0.873 ± 0.066**	**0.631 ± 0.064**	**1.68 ± 2.74**	**0.921 ± 0.063**	**0.943 ± 0.030**	**0.931 ± 0.044**
EGT Boundary-Focus	0.930 ± 0.045	0.872 ± 0.068	0.620 ± 0.067	1.76 ± 2.79	0.917 ± 0.065	0.946 ± 0.030	0.930 ± 0.045

**Table 5:** Detailed statistical comparison of models for Dice score.

Model	Baseline	Model Mean	Difference	Difference (%)	Wilcoxon *p*	Cohen’s *d*	Significance
Improved U-Net	0.9301	0.9312	+0.0011	+0.113%	0.0032	0.100	Yes
Fixed Multi-Loss	0.9301	0.9308	+0.0007	+0.076%	0.0037	0.092	Yes
EGT Standard	0.9301	0.9310	+0.0009	+0.097%	0.0032	0.100	Yes
EGT Aggressive	0.9301	0.9309	+0.0008	+0.088%	0.0144	0.070	Yes
EGT Boundary-Focus	0.9301	0.9297	−0.0004	−0.043%	0.7697	−0.038	No

**Table 6:** Detailed statistical comparison of models for Boundary Dice score.

Model	Baseline Mean	Model Mean	Difference	Difference (%)	Wilcoxon *p*	Cohen’s *d*	Significance
Improved U-Net	0.6276	0.6290	+0.0015	+0.235%	0.4657	0.065	No
Fixed Multi-Loss	0.6276	0.6235	−0.0040	−0.644%	0.0208	−0.185	Yes (↓)
EGT Standard	0.6276	0.6257	−0.0018	−0.291%	0.2226	−0.095	No
EGT Aggressive	0.6276	0.6311	+0.0036	+0.567%	0.0127	0.184	Yes (↑)
EGT Boundary-Focus	0.6276	0.6195	−0.0081	−1.283%	<0.001	−0.344	Yes (↓)

**Table 7:** Summary of statistical comparisons across additional metrics.

Metric	Improved	Fixed ML	EGT Std	EGT Agg	EGT BF
IoU	↑ 0.22%	↑ 0.17%	↑ 0.19%	↑ 0.17%	↓ 0.06%
HD95	↓ 1.81%	↑ 4.37%	↑ 6.62%	↑ 2.41%	↑ 7.73%
ASD	↑ 0.03%	↑ 4.91%	↑ 3.72%	↓ 0.13%	↑ 6.85%
Precision	↑ 0.04%	↓ 0.50%	↓ 0.26%	↓ 0.17%	↓ 0.69%
Recall	↑ 0.20%	↑ 0.69%	↑ 0.79%	↑ 0.40%	↑ 0.68%

**Note:** ↑ indicates improvement and ↓ indicates decline relative to the Baseline U-Net.

**Table 8:** Detailed weight evolution for EGT Aggressive.

Parameter	Initial	Final	Change	Change (%)
Dice Weight	0.350	0.101	−0.249	−71.1%
Focal Weight	0.350	0.292	−0.058	−16.5%
Boundary Weight	0.300	0.606	+0.306	+102.0%
Number of Generations	–	48	–	–

**Table 9:** Comparison of model training times.

Model	Time (s)	Time (min)	Relative to Baseline
Baseline U-Net	804.3	13.4	1.00×
Improved U-Net	1213.5	20.2	1.51×
Fixed Multi-Loss	1100.5	18.3	1.37×
EGT Standard	1251.8	20.9	1.56×
EGT Aggressive	1246.6	20.8	1.55×
EGT Boundary-Focus	1110.8	18.5	1.38×

**Table 10:** Cross-architecture comparison on the pediatric panoramic test set.

Model	Dice	IoU	ROC-AUC
EGT-UNet (Proposed)	0.931	0.874	0.994
U-Net (CNN Baseline)	0.930	0.872	0.995
UNet 3+ (Multi-Scale CNN)	0.929	0.870	0.995
Swin-UNet (Transformer)	0.907	0.833	0.992

**Table 11:** Summary of related studies in dental image segmentation and analysis.

Reference	Data Structure	Scan Type	Patient	Task	Model/Architecture	Dataset	Key Results
Hou et al. [5]	2D	Panoramic	Adult/General	Segmentation	Teeth U-Net (Enhanced U-Net)	1500 images	Accuracy: 98.53%; DSC: 94.28%
Mohan et al. [6]	2D	Panoramic (PXI)	Adult/General	Segmentation and Recognition	VGG-UNet + YOLO-V3	1000 images	Accuracy (Seg.): 98.84%; Accuracy (Rec.): 98%
Nader et al. [7]	2D	Panoramic	Adult/General	Segmentation	Modified U-Net (with Bounding Box Prior)	543 images	DSC: ~89.5% (vs. 85% for standard U-Net)
Tekin et al. [8]	2D	Bitewing	Adult/General	Segmentation	Mask R-CNN	1200 images	mAP (Seg.): 97.49%; mAP (Num.): 91.51%
Chandrashekar et al. [9]	2D	Panoramic	Adult/General	Segmentation and Identification	Collaborative (Mask R-CNN + Faster R-CNN)	Not specified	Accuracy (Seg.): 98.77%; Accuracy (ID): 98.44%
Gardiyanoğlu et al. [10]	2D	Panoramic (OPG)	Adult/General	Multi-object Segmentation	U-Net	8138 images	DSC (Teeth): 0.85; DSC (Caries): 0.88; DSC (Implants): 0.94
Sheng et al. [11]	2D	Panoramic	Adult	Segmentation	Swin-Unet (Transformer-based)	100 images	F1 Score: 0.6372; mIoU: 0.4689; Accuracy: 88.52%
Shaheen et al. [12]	3D	CBCT	Adult/General	Multi-class Segmentation and Classification	3D U-Net Pipeline	186 scans	Precision (Seg.): 98%; Recall (Class.): 98.5%
Kirnbauer et al. [13]	3D	CBCT	Adult/General	Periapical Lesion (PAL) Detection	Two-step (SCN + U-Net)	144 scans	Sensitivity: 97.1%; Specificity: 88.0%
Shen et al. [14]	3D	CBCT	Adult/General	Classification	Hybrid (CNN + Transformer)	554 samples	Accuracy: 91.3%; AUC: 99.7%
Wang et al. [15]	3D	CBCT	Adult/General	Segmentation	Trans-VNet (V-Net + Transformer)	Not specified	DSC (with artifacts): 88.67%; DSC (clear data): 96.44%
Li et al. [16]	3D	CBCT	Adult/General	Segmentation	Semantic Graph Attention (SGA) Network	350 scans	DSC: 91.13%; HD: 1.00 mm
Fontenele et al. [17]	3D	CBCT	Adult/General	Segmentation (with fillings)	3D U-Net	175 scans	IoU (with fillings): 0.91–0.95
Elsonbaty et al. [18]	3D	CBCT	Child	Primary Tooth Segmentation	CNN (3D U-Net)	37 scans (402 teeth)	Accuracy: 98%; DSC: 95%
Mladenovic et al. [19]	3D	CBCT	Child	Supernumerary Segmentation	Commercial 3D U-Net tools	1 case	N/A
Wang et al. [20]	3D Mesh	Intraoral Scanner (IOS)	Adult/General	Segmentation (robust to anomalies)	3D U-Net Pipeline	761 images	mIoU: 91.0%
Im et al. [21]	3D Mesh	Digital Dental Models	Adult/General	Tooth Segmentation	Dynamic Graph CNN (DGCNN)	516 models	Success Rate: 97.26%
Zhao et al. [22]	3D Mesh	Intraoral Scanner (IOS)	Adult/General	Segmentation	Graph Attentional Convolution (GAC) Network	80 models	OA: 94.84%; mIoU: 87.09%
Zheng et al. [23]	3D Mesh	3D Dental Models	Adult/General	Segmentation	TeethGNN (Graph Neural Network)	3828 models	mIoU: 97.37%; Accuracy: 98.89%
Lin et al. [24]	3D Point Cloud	Intraoral Scanner (IOS)	Adult/General	Segmentation	DBGANet (Dual-Branch GNN)	597 models	DSC: 94.48%
Zhuang et al. [25]	3D Point Cloud	3D Dental Models	Adult/General	Segmentation and Labeling	Hybrid (AlignNet + Multi-task Network)	Not specified	Practical Usability: 90.83%
Asci et al. [26]	2D	Panoramic	Child (4–14 yrs)	Caries Segmentation	U-Net	6075 images	F1 Score: 86.75%; Precision: 91.23%
Rubiu et al. [27]	2D	Panoramic	Child and Adult	Instance Segmentation (52 classes)	Mask R-CNN	1000 images	Accuracy: 98.4%; DSC: 0.87
Zhong et al. [28]	2D	X-ray	Child	Segmentation	GCNet (Grouped Attention)	Not specified	DSC: 0.9338; Sensitivity: 0.9426; Specificity: 0.9821
Zhang et al. [29]	2D	Panoramic	Child (2–13 yrs)	Caries Segmentation and Disease Detection	Tested U-Net, R2 U-Net, etc.	193 images	U-Net mIoU: 0.8387; U-Net Accuracy: 97.10%
Liu et al. [42]	2D	Panoramic (PAR)	Adult/General	Periodontitis Diagnosis	CNN	Not specified	Accuracy: 80.0%; AUC: 0.843
Cui et al. [43]	3D Point Cloud	3D Dental Models	Adult/General	Segmentation (robust to anomalies)	TSegNet (Two-stage network)	2000 models	DSC_point_: 98.0%; DSC_surface_: 98.6%
**Our Proposed Model**	**2D**	**Panoramic**	**Child (3–14 yrs)**	**Segmentation and Labeling**	**EGT-UNet**	**1269 images**	**DSC: 0.931, IoU: 0.873**

## Data Availability

The dataset used in this research includes anonymous pediatric tooth panoramic radiograph data collected from Ordu University Faculty of Dentistry. All images in the dataset were anonymized before use, and no personal information was kept. The anonymized dataset will be accessible from 15 July 2026 via the following GitHub repository: https://github.com/ulutashasan/pediatric_tooth_data. The ethical permission for this research was provided by the Ordu University Non-Interventional Scientific Research Ethics Committee (Permission Number: 223/18). The source code used in this study has been made publicly available at https://github.com/ulutashasan/pediatric_tooth_data.
